# Insomnia of students following the lifting of COVID-19 restrictions in China: Prevalence, influencing factors, and associations with depression, anxiety, and PTSD

**DOI:** 10.3389/fpsyt.2025.1680558

**Published:** 2025-12-08

**Authors:** Hang Zhang, Li Yin, Cong Wang, Yi-Hao Liu, Yi-Yue Yang, Lie Zhou, Hui Jin, Yun Xiao, Yang Wen, Jawad Ahmad, Jia Cai, Yu Wang, Lu Tan, Tao-Mei Li, Xue-Hua Huang, Jin Chen, Xiang-Dong Tang, Mao-Sheng Ran

**Affiliations:** 1 Mental Health Center, West China Hospital, Sichuan University, Chengdu, Sichuan, China; 2 Sleep Medicine Center, West China Hospital, Sichuan University, Chengdu, Sichuan, China; 3 National Center for Mental Disorders, West China Hospital, Sichuan University, Chengdu, Sichuan, China; 4 Department of Social Psychiatry, West China Hospital, Sichuan University, Chengdu, Sichuan, China; 5 Department of Clinical Epidemiology and Evidence-Based Medicine, West China Hospital, Sichuan University, Chengdu, Sichuan, China

**Keywords:** insomnia, influencing factors, mental health, youth, COVID-19

## Abstract

**Introduction:**

Insomnia has emerged as a major concern among youth following the lifting of coronavirus disease 2019 (COVID-19) restrictions in China. This study aimed to explore the prevalence and influencing factors of insomnia, as well as its associations with mental health, in young students during this period.

**Methods:**

A cross-sectional survey was conducted among 82,873 students in Sichuan Province, China, using a self-designed questionnaire and standardized assessment tools. Chi-square tests, ANOVA with *post-hoc* test analyses, and logistic regression were employed to identify the prevalence and influencing factors of insomnia and its associations with mental health.

**Results:**

A total of 28,178 (34.0%) students reported insomnia. Self-reported history of mental disorders, neglectful parenting, and initiating alcohol consumption during the pandemic were associated with higher odds of insomnia, whereas satisfaction with the academy, full recovery of daily routines, greatly improved family relationships, no change in friendships, and higher maternal education were associated with lower odds of insomnia (*p* < 0.001). Severe insomnia was strongly associated with depression, anxiety, and posttraumatic stress disorder (PTSD; ORs: 17.55, 3.35, and 17.45, respectively). Interaction effects were observed between mild to moderate insomnia and COVID-19 infection on depression, anxiety, and PTSD.

**Conclusion:**

Following the lifting of COVID-19 restrictions, insomnia remained prevalent among young Chinese students. Preexisting self-reported mental disorders, neglectful parenting, and pandemic-related maladaptive behaviors increase the risk of insomnia, whereas positive lifestyle adjustments, stable relationships, and higher maternal education appear protective. A significant association was observed between insomnia severity and symptoms of depression, anxiety, and PTSD. These findings highlight the persistent links among social, behavioral, and demographic factors, sleep, and mental health, emphasizing the importance of addressing sleep disturbances after the public health crisis.

## Introduction

Insomnia is a common sleep disorder characterized by difficulties in initiating or maintaining sleep, early awakening, and various daytime symptoms such as fatigue, emotional instability, and impaired cognitive function, which are significantly associated with both physical and mental health (1). The prevalence of insomnia in adults is estimated to range from 10% to 20%, with approximately 50% reporting a chronic course (2). Moreover, the prevalence in adolescents (18.5%) is equal to or higher than that in adults (3), with greater chronicity, as 88% of young students with a history of insomnia also report current symptoms (4).

The coronavirus disease 2019 (COVID-19) pandemic brings profound disruptions beyond its immediate health impact. Evidence indicates that the prevalence of insomnia symptoms during the early phase of the COVID-19 pandemic was roughly twice as high as prepandemic levels (5). Previous studies show that the relationship between COVID-19 and insomnia is complex and evolves with the progression of the pandemic (6). The 3P model of insomnia may provide a useful framework for understanding this dynamic. According to this model, insomnia develops through an interaction of predisposing, precipitating, and perpetuating factors (7). Women, individuals living in densely populated areas, those with a history of mental illnesses, or individuals with heightened concerns about COVID-19 are more likely to experience poor sleep quality, which may be regarded as a predisposing factor for insomnia (8–10). Pandemic-related stress may act as a precipitating factor, affecting sleep disturbances and maladaptive behaviors or beliefs, thereby perpetuating insomnia over time.

Insomnia has been linked to depression, anxiety, and suicidality (11). Among young students, the pandemic disrupted not only sleep patterns but also academic routines and lifestyles (12). Sleep insufficiency is closely associated with impaired emotional regulation, reduced positive affect, and emotional imbalance (13). Evidence also indicates an increasing prevalence of depression, anxiety, and posttraumatic stress disorder (PTSD) during the COVID-19 epidemic period, alongside a higher prevalence of insomnia (5, 14, 15). Nevertheless, most studies examining the link between insomnia and mental health problems were conducted before or during the early stages of the COVID-19 pandemic (16, 17). The association between insomnia and mental health problems after the lifting of COVID-19 restrictions in China remains unclear. Additionally, the small sample sizes of existing studies limit the analysis of odds ratios (ORs) for mental health problems across different levels of insomnia severity.

On 7 December 2022, China lifted restrictions on COVID-19. This period was characterized by a surge in the COVID-19 infection rates and an increase in the prevalence of insomnia (18). These changes were accompanied by shifts in lifestyles and social relationships, potentially influencing sleep and its association with mental health problems among young students. Therefore, this study aimed to investigate the prevalence and influencing factors of insomnia in young students following the lifting of COVID-19 restrictions in China, as well as to examine its associations with depression, anxiety, and PTSD. We also explored whether these associations differed by COVID-19 infection. We hypothesized that insomnia would be more prevalent following the lifting of COVID-19 restrictions and that it may be associated with social, behavioral, and demographic factors. Insomnia severity was expected to show positive associations with mental health symptoms, and these relationships might differ depending on COVID-19 infection status.

## Methods

### Study design and participants

Between 14 December 2022 and 28 February 2023, a cross-sectional study was conducted in Sichuan Province, China, to examine the prevalence and influencing factors of insomnia, as well as its relationship with other mental health symptoms. Sichuan Province is one of the most populous regions in China, with a large and diverse population. The study adopted a convenience sampling strategy at the school level. To ensure representativeness across different types of schools, invitations were sent to middle schools, high schools, colleges, and universities throughout Sichuan Province, and all procedures were carried out with the cooperation and oversight of these schools. Finally, 162 schools agreed to participate in the survey. All students in these schools were invited to join the survey without any restrictions. Subsequently, the online self-report questionnaires were distributed to school teachers or professors, who sent the questionnaires directly to their students via computer or telephone through the Wen Juanxing platform. Teachers only assisted in questionnaire distribution and had no access to individual responses, ensuring confidentiality and minimizing potential selection bias as much as possible. Informed consent was obtained online before the survey. This study was also approved by the Biomedical Research Ethics Committee of West China Hospital, Sichuan University (No. 2022-1790).

### Measures

Data were collected using online self-assessment questionnaires. All items were mandatory in the online system, preventing item-level missing data. Participants who did not complete the survey were excluded from the analysis. The questionnaire was divided into two parts. The first part comprised a self-designed survey, based on prior large-scale studies and reviewed by experts in psychology, psychiatry, sleep medicine, and public health, to explore the influencing factors of insomnia (19, 20). It included four main categories: (1) demographic characteristics, such as age, gender, education level, ethnicity, household registration, and self-reported personal and family history of mental disorders (based on the question, “Have you/your family members ever been diagnosed with or received treatment for a mental disorder?”); (2) family background, including household size, monthly family income, parents’ education level, and other relevant data; (3) COVID-19-related information, such as infection of the participant or family members, vaccination status, quarantine history, and psychological stress levels during different phase—phase 1: nationwide lockdown (1 January 2020 to 29 April 2020); phase 2: routine infection prevention and control (30 April 2020 to 6 December 2022); and phase 3: lifting of COVID-19 restrictions (after 7 December 2022); and (4) changes in life and relationships due to COVID-19 (details are listed in **Table 1**, **Supplementary Table S1**).

**Table 1:** Basic information of students with different severities of insomnia.

	No insomnia	Insomnia			*χ* ^2^		*P*
		Mild	Moderate	Severe			
Sex							
Male	24,580 (44.9)	8,044 (38.8)	2,006 (35.8)	756 (40.5)	350.14		< 0.001
Female	30,115 (55.1)	12,671 (61.2)	3,591 (64.2)	1,110 (59.5)			
Educational level							
Junior high school	18,587 (34.0)	4,115 (19.9)	1,092 (19.5)	363 (19.5)	1,918.33		< 0.001
Senior high school	21,687 (39.7)	10,505 (50.7)	2,910 (52.0)	1,009 (54.1)			
College and university	14,421 (26.4)	6,095 (29.4)	1,595 (28.5)	494 (26.5)			
History of mental disorders							
Yes	1,952 (3.6)	1,594 (7.7)	813 (14.5)	396 (21.2)	2,248.80		< 0.001
No	52,743 (96.4)	19,121 (92.3)	4,784 (85.5)	1,470 (78.8)			
Educational level of father							
Primary school and below	14,555 (26.6)	6,234 (30.1)	1,702 (30.4)	593 (31.8)	157.64		< 0.001
Middle school	24,944 (45.6)	9,197 (44.4)	2,542 (45.4)	794 (42.6)			
High school	10,089 (18.4)	3,629 (17.5)	929 (16.6)	311 (16.7)			
College and above	5,107 (9.3)	1,655 (8.0)	424 (7.6)	168 (9.0)			
Educational level of the mother							
Primary school and below	19,405 (35.5)	8,454 (40.8)	2,255 (40.3)	711 (38.1)	253.29		< 0.001
Middle school	22,240 (40.7)	8,097 (39.1)	2,181 (39.0)	733 (39.3)			
High school	8,800 (16.1)	2,878 (13.9)	818 (14.6)	286 (15.3)			
College and above	4,250 (7.8)	1,286 (6.2)	343 (6.1)	136 (7.3)			
Parenting style							
Authoritative	32,780 (59.9)	9,510 (45.9)	2,177 (38.9)	653 (35.0)	2,662.85		< 0.001
Authoritarian	10,870 (19.9)	5,542 (26.8)	1,652 (29.5)	529 (28.3)			
Neglectful	3,072 (5.6)	2,146 (10.4)	798 (14.3)	362 (19.4)			
Permissive	7,973 (14.6)	3,517 (17.0)	970 (17.3)	322 (17.3)			
Family economy							
Greatly worsened	3,760 (6.9)	1,820 (8.8)	746 (13.3)	410 (22.0)	1,652.89		< 0.001
Slightly worsened	21,627 (39.5)	9,813 (47.4)	2,649 (47.3)	789 (42.3)			
No change	13,532 (24.7)	3,763 (18.2)	860 (15.4)	215 (11.5)			
Slightly improved	6,968 (12.7)	2,332 (11.3)	551 (9.8)	157 (8.4)			
Greatly improved	1,062 (1.9)	281 (1.4)	83 (1.5)	30 (1.6)			
Not sure	7,746 (14.2)	2,706 (13.1)	708 (12.6)	265 (14.2)			
Family relationship							
Greatly worsened	1,347 (2.5)	720 (3.5)	373 (6.7)	276 (14.8)	3,161.84		< 0.001
Slightly worsened	4,804 (8.8)	3,229 (15.6)	1,153 (20.6)	389 (20.8)			
No change	33,115 (60.5)	10,746 (51.9)	2,431 (43.4)	650 (34.8)			
Slightly improved	8,115 (14.8)	3,297 (15.9)	843 (15.1)	236 (12.6)			
Greatly improved	2,976 (5.4)	701 (3.4)	168 (3.0)	67 (3.6)			
Not sure	4,338 (7.9)	2,022 (9.8)	629 (11.2)	248 (13.3)			
Friendship							
Greatly worsened	1,246 (2.3)	698 (3.4)	411 (7.3)	274 (14.7)	3,245.12		< 0.001
Slightly worsened	3,627 (6.6)	2,624 (12.7)	1,012 (18.1)	323 (17.3)			
No change	34,552 (63.2)	11,254 (54.3)	2,482 (44.3)	677 (36.3)			
Slightly improved	6,993 (12.8)	2,973 (14.4)	760 (13.6)	233 (12.5)			
Greatly improved	2,520 (4.6)	731 (3.5)	245 (4.4)	73 (3.9)			
Not sure	5,757 (10.5)	2,435 (11.8)	687 (12.3)	286 (15.3)			
Academic performance							
Progress	11,289 (20.6)	3,049 (14.7)	769 (13.7)	308 (16.5)	1,562.16		< 0.001
No change	22,892 (41.9)	7,294 (35.2)	1,760 (31.4)	531 (28.5)			
Regression	20,514 (37.5)	10,372 (50.1)	3,068 (54.8)	1,027 (55.0)			
Academic satisfactory							
Very satisfied	4,359 (8.0)	845 (4.1)	271 (4.8)	164 (8.8)	4,703.65		< 0.001
Satisfied	10,788 (19.7)	2,199 (10.6)	502 (9.0)	128 (6.9)			
Neutral	31,563 (57.7)	12,523 (60.5)	2,834 (50.6)	784 (42.0)			
Unsatisfied	6,843 (12.5)	4,213 (20.3)	1,428 (25.5)	472 (25.3)			
Very unsatisfied	1,142 (2.1)	935 (4.5)	562 (10.0)	318 (17.0)			
Academic recovery							
No	4,434 (8.1)	2,768 (13.4)	1,181 (21.1)	594 (31.8)	3,453.32		< 0.001
Part	36,554 (66.8)	15,331 (74.0)	3,804 (68.0)	1,072 (57.4)			
Total	13,707 (25.1)	2,616 (12.6)	612 (10.9)	200 (10.7)			
Influence on enrollment							
No	16,041 (29.3)	3,390 (16.4)	834 (14.9)	258 (13.8)	3,426.89		< 0.001
Minor	22,446 (41.0)	9,033 (43.6)	2,089 (37.3)	546 (29.3)			
Moderate	8,698 (15.9)	4,704 (22.7)	1,498 (26.8)	460 (24.7)			
Serious	2,009 (3.7)	1,175 (5.7)	593 (10.6)	347 (18.6)			
Not sure	5,501 (10.1)	2,413 (11.6)	583 (10.4)	255 (13.7)			
Influence on life routine							
No	26,594 (48.6)	7,098 (34.3)	1,665 (29.7)	536 (28.7)	1,899.88		< 0.001
Yes	28,101 (51.4)	13,617 (65.7)	3,932 (70.3)	1,330 (71.3)			
Recovery of life routine							
No	3,478 (6.4)	2,064 (10.0)	977 (17.5)	572 (30.7)	4,881.76		< 0.001
Part	31,060 (56.8)	14,803 (71.5)	3,814 (68.1)	1,041 (55.8)			
Total	20,157 (36.9)	3,848 (18.6)	806 (14.4)	253 (13.6)			
Smoking							
Never	53,363 (97.6)	19,693 (95.1)	5,158 (92.2)	1,652 (88.5)	958.91		< 0.001
Stop	327 (0.6)	292 (1.4)	115 (2.1)	61 (3.3)			
Always	804 (1.5)	531 (2.6)	236 (4.2)	116 (6.2)			
Start	201 (0.4)	199 (1.0)	88 (1.6)	37 (2.0)			
Drinking							
Never	50,505 (92.3)	17,903 (86.4)	4,513 (80.6)	1,373 (73.6)			< 0.001
Stop	2,592 (4.7)	1,629 (7.9)	528 (9.4)	200 (10.7)			
Always	1,167 (2.1)	807 (3.9)	388 (6.9)	202 (10.8)			
Start	431 (0.8)	376 (1.8)	168 (3.0)	91 (4.9)			
Exercising							
Yes	31,349 (57.3)	9,399 (45.4)	2,329 (41.6)	742 (39.8)		1,314.51	< 0.001
No	23,346 (42.7)	11,316 (54.6)	3,268 (58.4)	1,124 (60.2)			

Data are presented as *n* (%). No insomnia: ISI score ranges from 0 to 7; mild insomnia: ISI score ranges from 8 to 14; moderate insomnia: ISI score ranges from 15 to 21; and severe insomnia: ISI score ranges from 22 to 28.

Standardized scales were used in the second part of the questionnaire to assess insomnia severity and symptoms of depression, anxiety, and PTSD. The Insomnia Severity Index (ISI) is a commonly used seven-item scale that measures the severity of insomnia over the past 2 weeks (21). Based on the total score, insomnia severity was classified as no (0–7), mild (8–14), moderate (15–21), and severe (22–28). The Patient Health Questionnaire-9 (PHQ-9), comprising nine items, was used to assess possible depression symptoms over the past 2 weeks. Total scores ranged from 0 to 27, with a cutoff score of 5 indicating the presence of depression symptoms (22). The Generalized Anxiety Disorder seven-item scale (GAD-7) was used to evaluate anxiety symptoms over the past 2 weeks. A total score above 5 indicated the presence of anxiety symptoms in participants (23). PTSD symptoms in the past month, based on Diagnostic and Statistical Manual of Mental Disorders, Fifth Edition (DSM-5) diagnostic criteria, were measured using the Posttraumatic Stress Disorder Checklist for DSM-5 (PCL-5). The checklist includes 20 items with a total score ranging from 0 to 80 points, and a sum score above 33 was considered indicative of PTSD symptoms (24). The Cronbach’s α of ISI, PHQ-9, GAD-7, and PCL-5 was 0.93, 0.95, 0.97, and 0.98, respectively, indicating that all scales have stable reliability and validity in the Chinese population (25–27).

### Statistical analysis

**Figure 1:**
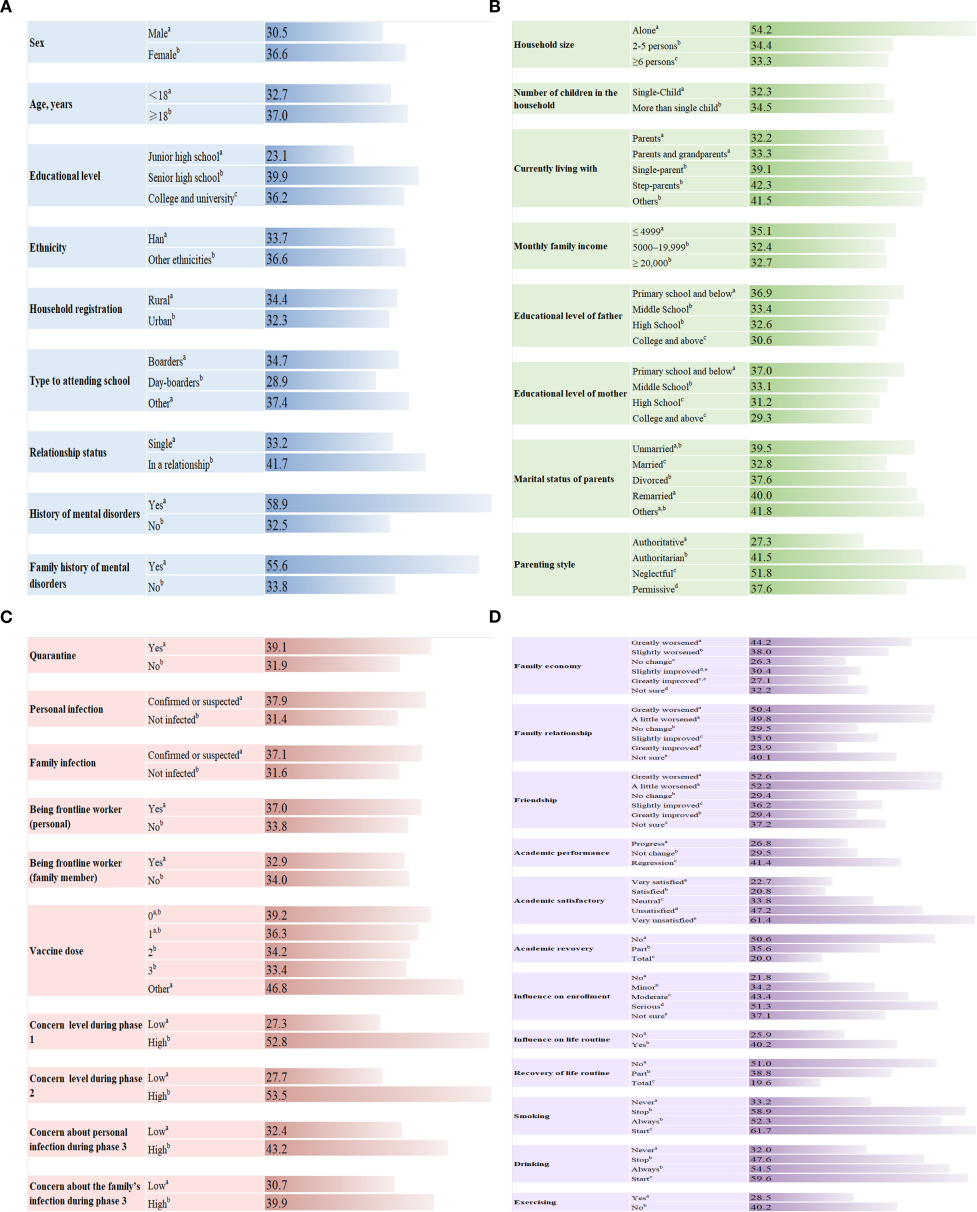
The prevalence of insomnia in subgroups. **(A)** Demographic characteristics. **(B)** Family background. **(C)** COVID-19-related information. **(D)** Changes in lifestyle and relationships. For groups with multiple items, each superscript letter (a–e) indicates a subset of the category, and the same superscript letter means insomnia proportions do not differ significantly from each other at the 0.05 level. Phase 1: the nationwide lockdown phase; phase 2: the routine infection prevention and control phase; phase 3: the lifting of COVID-19 restrictions phase.

Data are presented as mean ± standard deviation (SD) for continuous variables and as number (percentage) for categorical variables. The Chi-square test, with Bonferroni correction for multiple comparisons, was applied to categorical variables. ANOVA with *post-hoc* tests was used to compare differences among the four subgroups of insomnia severity. Logistic regression (forward selection, likelihood ratio) was employed to identify factors associated with insomnia. Spearman’s correlation analysis was conducted to examine the relationships between insomnia and other mental health symptoms. To evaluate the adjusted ORs and 95% confidence intervals (CI) for mental health problems across different levels of insomnia severity, logistic regression models were employed. Model 1 included all demographic characteristics, family background, COVID-19-related information, and changes in life and relationships listed in **Figure 1** as covariates. Model 2 further adjusted for other mental health problems; for example, when exploring the relationship between insomnia and symptoms of depression, it additionally adjusted for the other emotional symptoms (anxiety and PTSD) to account for potential confounding effects. To examine the interaction between insomnia severity and COVID-19 on mental health symptoms, both the main effects of insomnia severity and COVID-19, as well as their interaction term (insomnia severity × COVID-19), were included in the regression models. All statistical analyses were two-sided, with statistical significance set at *p* < 0.05. Data analysis was performed using SPSS version 25.0.

## Results

### Prevalence of insomnia symptoms

A total of 90,118 students agreed to participate, and 82,873 of them completed the questionnaire, yielding a response rate of 92.0%. Among the 82,873 participants, with a mean ± SD age of 16.1 years ± 2.5 years, 28,178 students reported insomnia symptoms, accounting for 34.0% of the total. Among students with insomnia, 20,715 (73.5%) had mild insomnia, 5,597 (19.9%) had moderate insomnia, and 1,866 (6.6%) had severe insomnia. Additionally, in the total sample, 32,322 (39.0%) reported difficulty falling asleep, 25,890 (31.2%) reported difficulty staying asleep, and 30,339 (36.6%) reported waking up too early. The average ISI score for participants was 5.80 ± 6.16.

The characteristics of participants with different insomnia severity levels are presented in **Table 1**, **Supplementary Table S1**. The prevalence of insomnia in subgroups is shown in **Figure 1**.

Women (36.6%), participants older than 18 years (37.0%), and senior high school students (39.9%) had significantly higher prevalence of insomnia symptoms. Participants with a personal history (58.9%) or a family history of mental disorders (55.6%) also showed higher prevalence of insomnia (all *p* < 0.05) (**Figure 1A**).

Regarding family background, the rates of insomnia were 30.6% and 29.3% among children whose parents’ educational level was college and above, and the prevalence of insomnia increased as the parental educational level decreased. Furthermore, a neglectful parenting style was associated with a higher prevalence of insomnia (51.8%) (**Figure 1B**).

Participants who expressed high concern about the COVID-19 pandemic during the nationwide lockdown phase (52.8%) and the routine infection prevention and control phase (53.5%) had a significantly higher prevalence of insomnia symptoms than those who did not express concern (*p* < 0.05). After the lifting of COVID-19 restrictions, the prevalence of insomnia remained higher among young students with high levels of concern (39.9% for concern about personal infection and 42.3% for concern about family infection, *p* < 0.05), although it was lower than in the early pandemic period (52.8% in phase 1 and 53.5% in phase 2) (**Figure 1C**).

Students whose family economy (44.2%), family relationships (50.4%), or friendships (52.6%) worsened, whose academic performance regressed (41.4%), or who felt very unsatisfied with academic performance (61.4%), had a higher prevalence of insomnia. Similarly, students whose enrollment (51.3%) or daily routines (40.2%) were seriously affected, whose academics (50.6%) or life routines (51.0%) had not recovered, or who started smoking or drinking during the pandemic and never exercised had a higher proportion of insomnia symptoms (61.7%, 59.6%, and 40.2%, respectively) (**Figure 1D**).

### Influencing factors of insomnia symptoms

**Figure 2:**
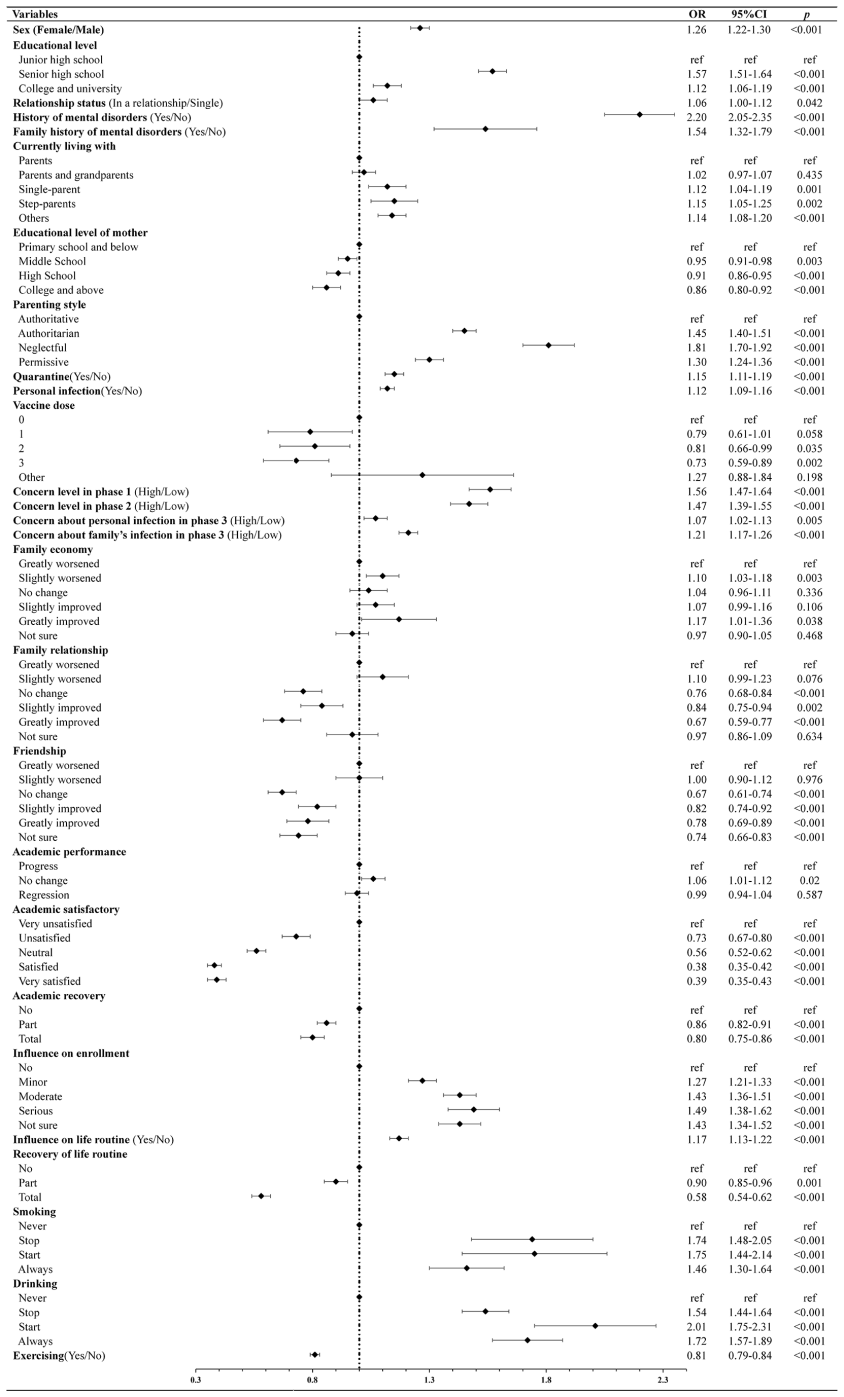
Factors influencing insomnia symptoms.

There were significant associations between insomnia and a personal history of mental disorders (OR = 2.20; 95% CI: 2.05–2.35; *p* < 0.001), a neglectful parenting style (OR = 1.81; 95% CI: 1.70–1.92; *p* < 0.001), and initiating alcohol consumption during the pandemic (OR = 2.01; 95% CI: 1.75–2.31; *p* < 0.001). Protective factors against insomnia included satisfaction with the academy (OR = 0.38; 95% CI: 0.35–0.42; *p* < 0.001), full recovery of daily routines (OR = 0.58; 95% CI: 0.54–0.62; *p* < 0.001), greatly improved family relationships (OR = 0.67; 95% CI: 0.59–0.77; *p* < 0.001), no change in friendships (OR = 0.67; 95% CI: 0.61–0.74; *p* < 0.001), and higher maternal education (OR = 0.86; 95% CI: 0.80–0.92; *p* < 0.001) (**Figure 2**).

### Associations between insomnia and mental health symptoms

**Figure 3:**
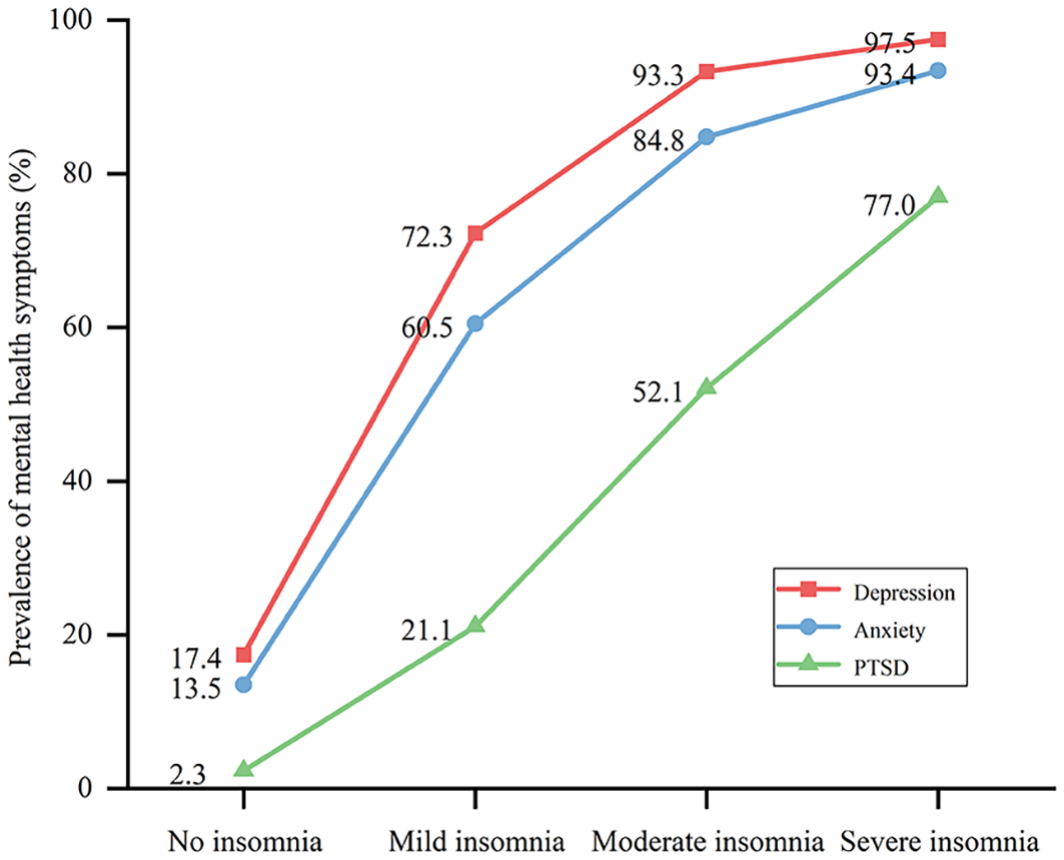
Prevalence of depression, anxiety, and posttraumatic stress disorder (PTSD) symptoms among students with/without different severities of insomnia.

In the whole sample, the prevalence of depression, anxiety, and PTSD symptoms was 38.1%, 31.8%, and 12.0%, respectively. Participants with more severe insomnia had higher scores on the PHQ-9 (19.70 ± 7.00), GAD-7 (15.31 ± 6.24), and PCL-5 (51.01 ± 24.17) (**Table 2**), and the prevalence of depression, anxiety, and PTSD symptoms among these students was 97.5%, 93.4%, and 77.0%, respectively (**Figure 3**). Moreover, the total score and the subscore of each item of the ISI were positively correlated with all the psychological scale scores (**Supplementary Figure S1**).

**Table 2:** Difference in mental health scores among varying severities of insomnia.

	Total	Insomnia				F	*P*
		No	Mild	Moderate	Severe		
PHQ-9	4.68 ± 6.03	1.99 ± 3.43	8.05 ± 5.37	13.42 ± 5.99	19.70 ± 7.00	26,660.17	< 0.001
GAD-7	3.39 ± 4.92	1.38 ± 2.83	5.80 ± 4.70	10.17 ± 5.52	15.31 ± 6.24	20,909.77	< 0.001
PCL-5	11.48 ± 16.21	4.99 ± 9.05	19.12 ± 15.52	33.39 ± 19.40	51.01 ± 24.17	19,870.23	< 0.001

Data presented as mean ± SD. *PHQ-9*, Patient Health Questionnaire-9; *GAD-7*, Generalized Anxiety Disorder seven-item scale; *PCL-5*, Posttraumatic Stress Disorder Checklist for DSM-5.

A significant association was observed between insomnia severity and symptoms of depression, anxiety, and PTSD after adjusting for demographic characteristics, COVID-19-related information, family background, and lifestyle changes in model 1 (*p* < 0.001). When further adjusted for other psychopathologies, insomnia severity remained significantly associated with depression (mild: OR = 4.43 [95% CI: 4.20–4.68]; moderate: OR = 11.93 [95% CI: 10.41–13.67]; severe: OR = 17.55 [95% CI: 12.35–24.95]), anxiety (mild: OR = 1.82 [95% CI: 1.73–1.93]; moderate: OR = 2.49 [95% CI: 2.24–2.75]; severe: OR = 3.35 [95% CI: 2.67–4.21]), and PTSD (mild: OR = 2.84 [95% CI: 2.63–3.06]; moderate: OR = 7.41 [95% CI: 6.77–8.11]; severe: OR = 17.45 [95% CI: 15.18–20.06]) (*p* < 0.001) (**Table 3**).

**Table 3:** Adjusted odds ratios (ORs) of mental health symptoms across different severities of insomnia.

	Insomnia severity	Model 1		Model 2	
		OR (95%CI)	*p*	OR (95%CI)	*p*
Depression	No	Ref		Ref	
	Mild	9.26 (8.90-9.63)	<0.001	4.43 (4.20-4.68)	<0.001
	Moderate	40.44 (36.24-45.13)	<0.001	11.93 (10.41-13.67)	<0.001
	Severe	96.43 (71.65-129.98)	<0.001	17.55 (12.35-24.95)	<0.001
Anxiety	No	Ref		Ref	
	Mild	7.31 (7.03-7.61)	<0.001	1.82 (1.73-1.93)	<0.001
	Moderate	21.97 (20.28-23.81)	<0.001	2.49 (2.24-2.75)	<0.001
	Severe	46.40 (38.42-56.04)	<0.001	3.35 (2.67-4.21)	<0.001
PTSD	No	Ref		Ref	
	Mild	8.79 (8.20-9.42)	<0.001	2.84 (2.63-3.06)	<0.001
	Moderate	29.99 (27.59-32.59)	<0.001	7.41 (6.77-8.11)	<0.001
	Severe	74.82 (65.66-85.25)	<0.001	17.45 (15.18-20.06)	<0.001

Model 1: adjusted for all demographic characteristics, family background, COVID-19-related information, and the impact of COVID-19 on lifestyles and behaviors, without the insomnia group as the reference. Model 2: binary logistic regression adjusted for all covariates in model 1 and further adjusted for other psychopathologies, without the insomnia group as the reference.

*PTSD*, posttraumatic stress disorder.

### Interaction effect between COVID-19 infection and insomnia on different mental health symptoms

In further regression models, we included insomnia severity, COVID-19 infection, and their interaction terms to examine the interaction effect on mental health symptoms. Insomnia severity remained significantly associated with mental health symptoms (*p* < 0.05). COVID-19 infection alone showed only weak associations with depression and anxiety symptoms. Weak interaction effects between insomnia severity and COVID-19 infection were observed for mild and moderate insomniaxon depression, anxiety, and PTSD symptoms (*p* < 0.01) (**Table 4**).

**Table 4:** Main and interaction effects of insomnia and COVID-19 infection on mental health.

	Depression		Anxiety		PTSD	
	OR (95% CI)	*P*	OR (95% CI)	*P*	OR (95% CI)	*P*
Insomnia severity						
No	Ref		Ref		Ref	
Mild	9.89 (9.39–10.42)	< 0.001	8.05 (7.64–8.48)	< 0.001	9.64 (8.82–10.53)	< 0.001
Moderate	45.94 (39.59–53.31)	< 0.001	24.88 (22.33–27.73)	< 0.001	33.32 (29.87–37.16)	< 0.001
Severe	105.31 (71.44–155.24)	< 0.001	52.83 (41.10–67.91)	< 0.001	79.23 (66.54–94.33)	< 0.001
COVID-19 infection						
No	Ref		Ref		Ref	
Yes	1.07 (1.01–1.15)	0.033	1.15 (1.07–1.23)	< 0.001	1.13 (0.99–1.29)	0.069
Insomnia severity * COVID-19 infection						
No * No infection	Ref		Ref		Ref	
Mild * Infection	0.86 (0.79–0.93)	< 0.001	0.80 (0.74–0.87)	< 0.001	0.79 (0.69–0.91)	0.001
Moderate * Infection	0.75 (0.60–0.93)	0.008	0.75 (0.64–0.88)	< 0.001	0.77 (0.66–0.91)	0.002
Severe * Infection	0.80 (0.44–1.47)	0.477	0.74 (0.50–1.07)	0.111	0.86 (0.66–1.11)	0.239

## Discussion

In this large sample cross-sectional study, a high prevalence of insomnia (34%), depression (38.1%), anxiety (31.8%), and PTSD (12%) symptoms was observed among young students following the lifting of COVID-19 restrictions in China. Self-reported history of mental disorders, neglectful parenting styles, and pandemic-related maladaptive behaviors were associated with an increased risk of insomnia, whereas positive lifestyle adjustments, stable relationships, and higher maternal education appeared protective. Significant associations were also identified between insomnia severity and symptoms of depression, anxiety, and PTSD, even after adjusting for demographic covariates and other mental health symptoms. Additionally, weak interaction effects were observed between mild to moderate insomnia and COVID-19 infection on depression, anxiety, and PTSD symptoms.

### Prevalence of insomnia symptoms

In this study, the prevalence of insomnia (34.0%) was notably higher than reports from China during the early pandemic period of COVID-19 in 2020 (16.9%) (28). As the epidemic progressed, the psychological and academic pressures increased, accompanied by a rising prevalence of insomnia. The observed variation in insomnia prevalence across different periods may reflect the long-term mental health impact of the epidemic, particularly due to disruptions to biological rhythms and the cumulative effects of stressors, such as increased media exposure to COVID-19 infection and heightened perceived susceptibility and persistent fear of COVID-19 after the lifting of COVID-19 restrictions in China (29). Epidemiological evidence also showed that mental health symptoms increased during recurrent outbreaks of COVID-19 and persisted after the emergency status ended (30). These findings suggest that the psychological impact of the pandemic extends beyond its acute phase, potentially causing functional impairment for individuals and exerting sustained pressure on community mental health services.

### Predisposing factors of insomnia

Three main influencing factors of insomnia following the lifting of COVID-19 restrictions in China can be considered potential predisposing factors of insomnia during this period. The first factor was a self-reported history of mental disorders. In this study, the prevalence of insomnia among participants with a history of mental disorders was 58.9%, nearly double that of participants without such a history, highlighting the significant role of mental health history (31). Although biological mechanisms could not be identified in this study, previous research suggests that alterations in neurotransmitters such as noradrenaline (NA) and orexin (both of which play crucial roles in regulating the sleep–wake cycle and stress response) may contribute to the observed associations (32, 33). Second, neglectful parenting, characterized by low support and limited behavioral control of children (34), was associated with a higher prevalence of insomnia. Parents with this parenting style may struggle to help their children develop adaptive emotional regulation or maintain consistent sleep routines, potentially increasing susceptibility to sleep problems under stress (35). Third, parental education level, especially maternal education, showed a negative association with insomnia. This may be related to the central role of mothers in child-rearing, as they often take greater responsibility for caregiving and emotional support (36). Mothers with higher educational levels tend to have greater coping strategies and communication skills, promote healthier sleep routines and lifestyles in their children, and demonstrate increased sensitivity to their children’s stress. These characteristics may enable them to respond more effectively to their children’s emotional needs and reduce the risk of insomnia through effective communication and emotional support (37).

### Precipitating and perpetuating factors of insomnia

Changes in lifestyle and relationships appeared to play a precipitating and perpetuating role in sleep disturbances. In this study, we found that the prevalence of insomnia symptoms was higher in young students who drank or smoked than in those who did not. Alcohol and nicotine are well-established risk factors for insomnia, as they disrupt the electrophysiological structure of sleep, affect biological rhythms, and modulate various neurotransmitter systems (38–40). Moreover, participants who initiated drinking or smoking during the COVID-19 pandemic exhibited a higher prevalence of insomnia compared to those who maintained their prepandemic drinking and smoking habits. This suggests that although unhealthy lifestyles are associated with sleep disturbances, the initiation of such behaviors has a more pronounced impact on the prevalence of insomnia.

Furthermore, disruptions of lifestyle and social relationships during the pandemic were associated with a higher likelihood of insomnia, whereas the restoration of these factors following the lifting of COVID-19 restrictions may be linked to a lower prevalence of insomnia. Specifically, disturbances in daily routines and academic activities during the pandemic were associated with more severe insomnia symptoms, while the recovery of daily routines was linked to better sleep. A similar pattern was observed for other psychosocial factors: improvements in family relationships, stable friendships, and higher levels of academic satisfaction were associated with a lower prevalence of insomnia. Previous studies did not report significant behavioral changes or improvements in mental health during the spring of 2021, which may be attributed to the ongoing global nature of the COVID-19 pandemic at that time (41).

### Association between insomnia and mental health problems

Significant associations were found between insomnia severity and symptoms of depression, anxiety, and PTSD, even after adjusting for covariates—including all the demographic characteristics, family background, COVID-19-related information, and the impact of COVID-19 on lifestyles and behaviors—as well as other mental health symptoms. These findings are consistent with previous evidence showing that individuals with insomnia tend to report higher levels of emotional distress. Before the outbreak of COVID-19, studies indicated that young women with insomnia had higher odds of depression (OR = 2.6–4.4) and anxiety (OR = 2.4–2.9) (42). Another meta-analysis showed that insomnia increased the risk of depression (OR = 2.83 [95% CI: 1.55–5.17]) and anxiety (OR = 3.23 [95% CI: 1.52–6.85]) (43). Previous studies have reported correlations between insomnia and mental health problems (17, 44). Strong associations were also observed in this study between insomnia severity and symptoms of depression or PTSD, even after further adjusting for the influence of other mental health symptoms. The COVID-19 pandemic, as a large-scale public health stressor, may contribute to the link between insomnia and emotional distress. Previous research suggests that chronic sleep disturbance can alter corticolimbic circuits involved in emotional regulation, fear processing, and cognitive control (42, 45, 46). Repeated exposure to stress and insufficient sleep has been suggested to cause physiological arousal and dysregulation of neural pathways involved in mental health (47, 48). Although biological mechanisms could not be examined in the present study, these previous studies may partly explain the observed relationship between insomnia and symptoms of depression, anxiety, or PTSD after the lifting of COVID-19 restrictions in China.

This study was conducted after the lifting of COVID-19 restrictions in China, following a nationwide surge in infection rates. Accordingly, the potential interaction between COVID-19 infection and insomnia severity on mental health symptoms was examined. Although the interaction effect reached statistical significance for mild to moderate insomnia with COVID-19 infection, the observed effect was modest and appeared weaker than the individual associations of insomnia or infection with depression and anxiety. One explanation is that individuals with mild to moderate insomnia may not experience the persistent hyperarousal observed in those with severe insomnia (49). In these individuals, COVID-19 infection may trigger adaptive stress responses, helping them stay alert or cope more effectively. This is consistent with the inverted U-shaped stress model, which suggests that moderate stress can be beneficial, while excessive stress may impair emotional balance (50–52).

Accumulating evidence indicates that the COVID-19 pandemic has contributed to a persistent global mental health burden (53–55). Integrating mental health into primary care and implementing long-term monitoring of mental health symptoms may facilitate early intervention (53). Coordinated and sustained efforts to provide accessible sleep-related interventions will be crucial in the postpandemic era.

### Strengths and limitations

This study has several strengths and limitations. The large sample of young students reduces study bias and enhances statistical power, as well as the robustness of the findings. By examining the mental health impacts of insomnia in young students during the post-COVID-19 restriction period, the study provides valuable insights into the lasting effects of the pandemic on insomnia and mental health problems, and offers an improved understanding of mental health risks during the recovery phase. However, some limitations should be considered when interpreting the results. First, due to the cross-sectional design, the study can only provide risk associations, and causal relationships cannot be inferred. Second, this study was conducted only in Sichuan Province, which may limit the generalizability of the findings to other regions. Third, all data were self-reported, which may introduce recall or reporting bias and potentially lead to overestimation of prevalence and associations. Although COVID-19-related symptoms were included, information on infection timing and severity, as well as other comorbid health conditions, was not assessed, which may result in residual confounding. Finally, participants with incomplete or invalid responses were excluded from the analyses; potential bias due to nonrandom missing data cannot be entirely ruled out.

Further prospective, multicenter, longitudinal studies across China are needed to generalize the findings and clarify the potential mechanisms underlying the relationships among influencing factors, insomnia, and mental health problems. In addition, future studies should include clinical interviews or objective measures to validate the results.

## Conclusion

This cross-sectional study with a large sample size was conducted after the lifting of the COVID-19 restrictions in China. The findings showed that the prevalence of insomnia symptoms in young students during this special period was significantly associated with a history of mental disorders, neglectful parenting styles, maternal education levels, and changes in lifestyle and relationships. Moreover, significant associations were observed between insomnia and symptoms of depression, anxiety, and PTSD. In addition, mild interaction effects were noted between mild to moderate insomnia and COVID-19 infection on depression, anxiety, and PTSD symptoms.

These findings suggest that even after the lifting of COVID-19 restrictions, insomnia remains prevalent among students and is influenced by individual, family, pandemic-related, and psychosocial factors. The severity of insomnia is closely associated with symptoms of depression, anxiety, and PTSD, reflecting the prolonged psychological burdens following major public health crises and highlighting the importance of providing psychosocial support to young students during the recovery period. School-based psychological screening, expanded access to digital sleep interventions, and strengthened primary-care referral pathways may help reduce mental health burdens. Targeted interventions addressing modifiable factors, such as parenting styles, lifestyle adjustments, and relationship stability, may help reduce the occurrence and severity of insomnia, as well as other mental health problems, in this population.

## Data Availability

The data are available on reasonable request to the corresponding author. Requests to access these datasets should be directed to msrancd@outlook.com.
