# Supplementary material for: Insomnia of students following the lifting of COVID-19 restrictions in China: Prevalence, influencing factors, and associations with depression, anxiety, and PTSD
Source: Front Psychiatry. 2025 Dec 8;16:1680558. doi: 10.3389/fpsyt.2025.1680558 (PMC12719473; doi:10.3389/fpsyt.2025.1680558)
Supplement: Supplementary file 1 [file Table1.docx]

**Table S1. The supplement basic information of students with different insomnia severities**

|  | **No** | **Insomnia** | | | **X^2^** | ***p*** |
| --- | --- | --- | --- | --- | --- | --- |
|  | **insomnia** | **Mild** | **Moderate** | **Severe** |  |  |
| **Age, years** |  |  |  |  | 152.20 | <0.001 |
| <18 | 38520 (70.4) | 13707 (66.2) | 3698 (66.1) | 1283 (68.8) |  |  |
| ≥18 | 16175 (29.6) | 7008 (33.8) | 1899 (33.9) | 583 (31.2) |  |  |
| **Ethnicity** |  |  |  |  | 43.16 | <0.001 |
| Han | 48779 (89.2) | 18215 (87.9) | 4869 (87.0) | 1674 (89.7) |  |  |
| Other ethnicities | 5916 (10.8) | 2500 (12.1) | 728 (13.0) | 129 (10.3) |  |  |
| **Household registration** |  |  |  |  | 27.80 | <0.001 |
| Rural | 44456 (81.3) | 17139 (82.7) | 4639 (82.9) | 1506 (80.7) |  |  |
| Urban | 10239 (18.7) | 3576 (17.3) | 958 (17.1) | 360 (19.3) |  |  |
| **Relationship status** |  |  |  |  | 278.64 | <0.001 |
| Single | 50120 (91.6) | 18448 (89.1) | 4881 (87.2) | 1579 (84.6) |  |  |
| In a relationship | 4575 (8.4) | 2267 (10.9) | 716 (12.8) | 287 (15.4) |  |  |
| **Family history of mental disorders** | |  |  |  | 245.91 | <0.001 |
| Yes | 372 (0.7) | 284 (1.4) | 124 (2.2) | 57 (3.1) |  |  |
| No | 54323 (99.3) | 20431 (98.6) | 5473 (97.8) | 1809 (96.9) |  |  |
| **Type to attending school** |  |  |  |  | 158.20 | <0.001 |
| Boarders | 46618 (85.2) | 18256 (88.1) | 4955 (88.5) | 1599 (85.7) |  |  |
| Day-boarders | 7630 (14.0) | 2263 (10.9) | 597 (10.7) | 241 (12.9) |  |  |
| Other | 447 (0.8) | 196 (0.9) | 45 (0.8) | 26 (1.4) |  |  |
| **Household size** |  |  |  |  | 31.62 | <0.001 |
| Alone | 22 (0.04) | 15 (0.1) | 8 (0.1) | 3 (0.2) |  |  |
| 2-5 persons | 33610 (61.45) | 12884 (62.2) | 3514 (62.8) | 1220 (65.4) |  |  |
| ≥6 persons | 21063 (38.51) | 7816 (37.7) | 2075 (37.1) | 643 (34.5) |  |  |
| **Number of children in the household** | |  |  |  | 40.42 | <0.001 |
| Single-Child | 13852 (25.3) | 4790 (23.1) | 1355 (24.2) | 474 (25.4) |  |  |
| More than single child | 40843 (74.7) | 15925 (76.9) | 4242 (75.8) | 1392 (74.6) |  |  |
| **Monthly family income** |  |  |  |  | 106.43 | <0.001 |
| ≤ 4999 | 31441 (57.5) | 12462 (60.2) | 3400 (60.7) | 1148 (61.5) |  |  |
| 5000–19,999 | 20750 (37.9) | 7415 (35.8) | 1946 (34.8) | 588 (31.5) |  |  |
| ≥ 20,000 | 2504 (4.6) | 838 (4.0) | 251 (4.5) | 130 (7.0) |  |  |
| **Currently living with** |  |  |  |  | 521.97 | <0.001 |
| Parents | 36770 (67.2) | 13051 (63.0) | 3330 (59.5) | 1070 (57.3) |  |  |
| Parents and grandparents | 8660 (15.8) | 3207 (15.5) | 836 (14.9) | 4317 (14.7) |  |  |
| Single-parent | 2947 (5.4) | 1356 (6.5) | 398 (7.1) | 136 (7.3) |  |  |
| Step-parents | 1655 (3.0) | 813 (3.9) | 292 (5.2) | 108 (5.8) |  |  |
| Others | 4663 (8.5) | 2288 (11.0) | 741 (13.2) | 278 (14.9) |  |  |
| **Marital status of parents** |  |  |  |  | 320.80 | <0.001 |
| Unmarried | 857 (1.6) | 385 (1.9) | 113 (2.0) | 62 (3.3) |  |  |
| Married | 45059 (82.4) | 16415 (79.2) | 4274 (76.4) | 1342 (71.9) |  |  |
| Divorced | 4713 (8.6) | 2017 (9.7) | 602 (10.8) | 220 (11.8) |  |  |
| Remarried | 3438 (6.3) | 1569 (7.6) | 514 (9.2) | 213 (11.4) |  |  |
| Other | 628 (1.1) | 329 (1.6) | 94 (1.7) | 29 (1.6) |  |  |
| **Family infection** |  |  |  |  | 289.25 | <0.001 |
| Confirmed or suspected cases | 22891 (41.9) | 9827 (47.4) | 2774 (49.6) | 910 (48.8) |  |  |
| Not infected | 31804 (58.1) | 10888 (52.6) | 2823 (50.4) | 956 (51.2) |  |  |
| **Being frontline worker (personal)** | |  |  |  | 25.28 | <0.001 |
| Yes | 2628 (4.8) | 1092 (5.3) | 342 (6.1) | 111 (5.9) |  |  |
| No | 52067 (95.2) | 19623 (94.7) | 5255 (93.9) | 1755 (94.1) |  |  |
| **Being frontline worker (family member)** | |  |  |  | 7.80 | 0.050 |
| Yes | 1999 (3.7) | 689 (3.3) | 225 (4.0) | 67 (3.6) |  |  |
| No | 52696 (96.3) | 20026 (96.7) | 5372 (96.0) | 1799 (96.4) |  |  |
| **Quarantine** |  |  |  |  | 443.22 | <0.001 |
| No | 39948 (73.0) | 13941 (67.3) | 3595 (64.2) | 1155 (61.9) |  |  |
| Yes | 14747 (27.0) | 6774 (32.7) | 2002 (35.8) | 711 (38.1) |  |  |
| **Personal infection** |  |  |  |  | 397.69 | <0.001 |
| Confirmed or suspected cases | 20683 (37.8) | 9136 (44.1) | 2634 (47.1) | 861 (46.1) |  |  |
| Not infected | 34012 (62.2) | 11579 (55.9) | 2963 (52.9) | 1005 (53.9) |  |  |
| **Vaccine dose** |  |  |  |  | 83.84 | <0.001 |
| 0 | 299 (0.5) | 123 (0.6) | 41 (0.7) | 29 (1.6) |  |  |
| 1 | 571 (1.0) | 219 (1.1) | 65 (1.2) | 41 (2.2) |  |  |
| 2 | 35615 (65.1) | 13557 (65.4) | 3705 (66.2) | 1234 (66.1) |  |  |
| 3 | 18103 (33.1) | 6746 (32.6) | 1771 (31.6) | 553 (29.6) |  |  |
| Other | 107 (0.2) | 70 (0.3) | 15 (0.3) | 9 (0.5) |  |  |
| **Concern level in phase 1** |  |  |  |  | 5071.86 | <0.001 |
| Low level | 44452 (81.3) | 12930 (62.4) | 2917 (52.1) | 859 (46.0) |  |  |
| High level | 10243 (18.7) | 7785 (37.6) | 2680 (47.9) | 1007 (54.0) |  |  |
| **Concern level in phase 2** |  |  |  |  | 4907.10 | <0.001 |
| Low level | 45262 (82.8) | 13319 (64.3) | 3092 (55.2) | 917 (49.1) |  |  |
| High level | 9433 (17.2) | 7396 (35.7) | 2505 (44.8) | 949 (50.9) |  |  |
| **Concern about personal infection in phase 3** | |  |  |  | 776.22 | <0.001 |
| Low level | 47573 (87.0) | 17093 (82.5) | 4313 (77.1) | 1348 (72.2) |  |  |
| High level | 7122 (13.0) | 3622 (17.5) | 1284 (22.9) | 518 (27.8) |  |  |
| **Concern about the family’s infection in phase 3** | | |  |  | 834.73 | <0.001 |
| Low level | 37060 (67.8) | 12476 (60.2) | 3026 (54.1) | 951 (51.0) |  |  |
| High level | 17635 (32.2) | 8239 (39.8) | 2571 (45.9) | 915 (49.0) |  |  |

**Notes:** Data presented as n (%). No insomnia, ISI score ranges from 0 to 7; mild insomnia, ISI score ranges from 8 to 14; moderate insomnia, ISI score ranges from 15 to 21 and severe insomnia, ISI score ranges from 22 to 28.


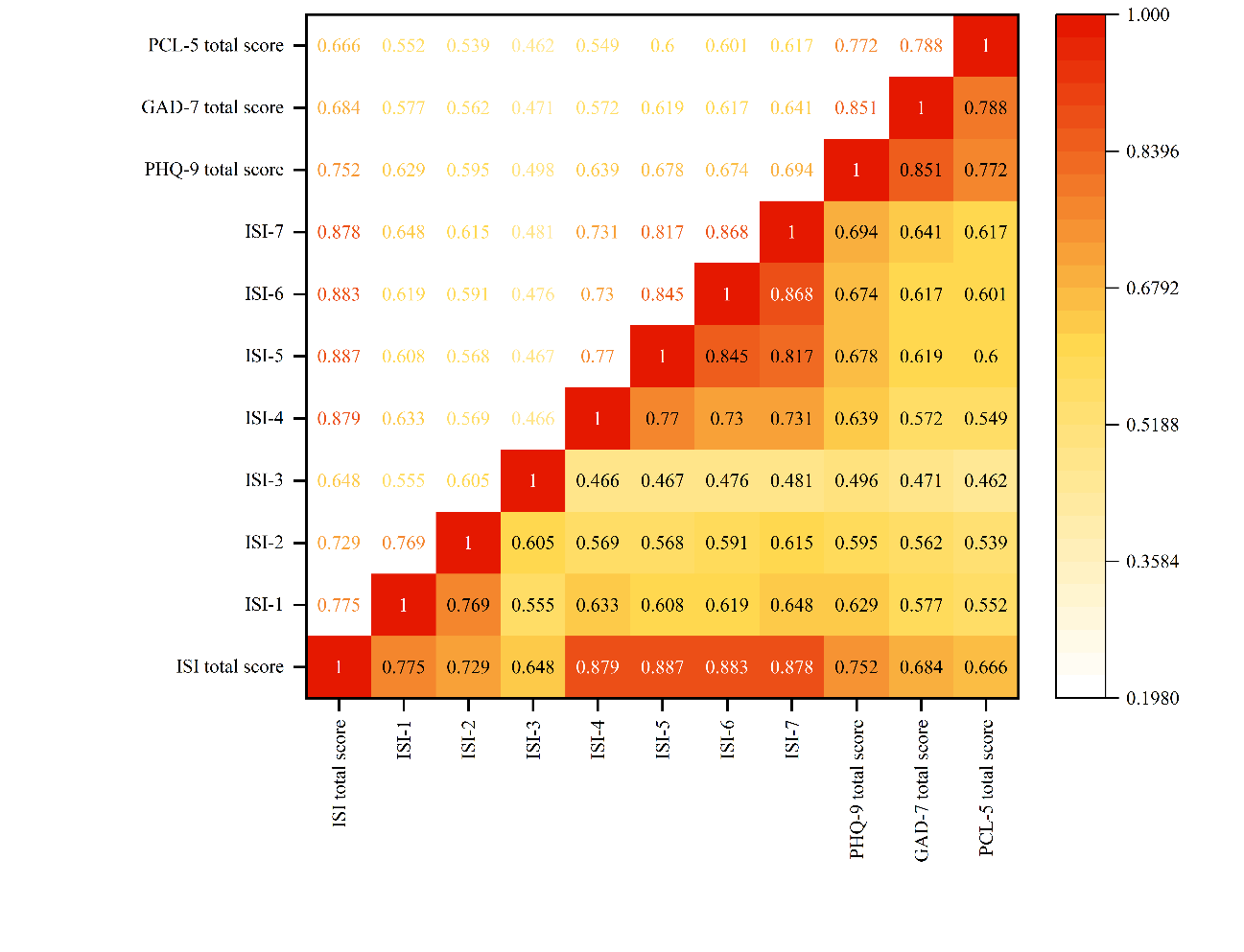


**Fig.S1 Correlations between the ISI scores and different psychological scale scores in total sample**
